# Impact of Obesity Phenotype on Central Aortic Hemodynamics and Arterial Stiffness in a Chinese Health Assessment Population

**DOI:** 10.31083/j.rcm2306216

**Published:** 2022-06-15

**Authors:** Huijuan Chao, Yueliang Hu, Qian Wang, Biwen Tang, Audrey Adji, Alberto Avolio, Kun Qian, Junli Zuo

**Affiliations:** ^1^Department of Geriatrics, Ruijin Hospital, Shanghai Jiao Tong University School of Medicine, 200025 Shanghai, China; ^2^Department of Biomedical Sciences, Faculty of Medicine, Health and Human Sciences, Macquarie University, 2109 Sydney, Australia; ^3^Faculty of Medicine, University of New South Wales, 2033 Sydney, Australia; ^4^St Vincent’s Clinical Campus, University of New South Wales Medicine and Health, 2010 Sydney, Australia

**Keywords:** obesity, central aortic hemodynamics, arterial stiffness, Chinese population

## Abstract

**Background:**

This study aimed to explore the association between BMI and/or central obesity parameters and measures of arterial hemodynamics to assess the effect of obesity on function of large arteries.

**Methods:**

Data was obtained from 634 subjects undergoing health assessment at Ruijin Hospital, Shanghai. Subjects were divided into 3 groups according to their Body Mass Index (BMI (kg/$m^{2}$) <24 normal, 24–28 overweight, ≥28 obese). In addition, central obesity was described by waist-hip ratio (WHR) and waist-height ratio (WHtR). Radial arterial waveforms and carotid-femoral pulse wave velocity (cf-PWV) were measured with the subjects recumbent. Central arterial pressures were measured by pulse wave analysis of the radial waveform calibrated to peripheral cuff systolic (PSP) and diastolic pressure (PDP) to obtain central systolic pressure (CSP), central diastolic pressure (CDP), central pulse pressure (CPP), central augmentation pressure (CAP), and central augmentation index (cAIx). Pulse pressure was determined from the ratio of peripheral (PPP) and central (CPP) pulse pressure (PPP/CPP).

**Results:**

CAP and cAIx were lowest in the obese group (*p *< 0.01). Pressure amplification was significantly higher as BMI increased (*p <* 0.05). After adjusting for confounding factors, WC, WHtR and WHR were independent risk factors for cf-PWV (β = 0.120, *p* = 0.001, β = 0.103, *p *= 0.004, β = 0.092, *p* = 0.013), When BMI, WC, WHtR, WHR were put into the stepwise linear regression model, only WC was an independent risk factor for cf-PWV (β = 0.135, *p *< 0.001).

**Conclusions:**

Central obesity (WC and WHR) measures may have greater predictive value for vascular stiffness than BMI. This possibility warrants further studies focused on arterial wave travel and its relationship with body fat distribution.

## 1. Introduction 

Obesity is a risk factor for all-cause and cardiovascular mortality. Previous studies have confirmed that obesity increases the incidence of cardiovascular events [1, 2]. However, some long-term follow-up studies have suggested a negative correlation between body mass index (BMI) and prognosis of cardiovascular disease and target organ damage, known as the “obesity paradox” [3, 4]. The obesity paradox may be caused by the confounding of research results by a variety of factors, such as BMI. BMI, one of the most frequently used surrogate anthropometric measures for obesity, does not distinguish between muscle and fat, and poorly reflects body fat distribution [5, 6]. The value of BMI in assessing and diagnosing obesity has been questioned.

Central aortic pressure has been suggested to provide information regarding end-organ damage additional to that provided by conventional brachial artery pressure. Prospective studies have found that central blood pressure can predict vascular events better than peripheral blood pressure, and that it is closely related to cardiovascular and cerebrovascular endpoint events [7, 8]. On the other hand, changes related to damage of vascular structure and function are independent risk factors for the occurrence and development of cardiovascular events and may be better indicators or alternative endpoints for the prediction of cardiovascular risk [7, 9]. Pulse wave velocity (PWV) is currently recognized as the best indicator for noninvasive detection of arterial stiffness, which can effectively reflect functional changes. According to the latest European Society of Hypertension expert consensus on carotid-femoral PWV (cf-PWV) for clinical practice, the cut-off value of cf-PWV for predicting cardiovascular events has been found to be 10 m/s [10].

This study aims to explore the association between different obesity phenotypes and central aortic hemodynamics and vascular stiffness so as to explore a more reasonable way to evaluate the clinical significance of obesity in cardiovascular events and to guide the individualized treatment of obesity.

## 2. Materials and Methods

### 2.1 Study Population

A total of 653 participants who received a routine physical examination at Ruijin Hospital from December 2017 to December 2019 were recruited. Nineteen cases were excluded due to missing data. Finally, 634 cases were included in this study. The inclusion criteria were age ≥18 years old, agreement to participate in this study and written informed consent. Exclusion criteria were: (1) Acute and serious heart disease (NewYork Heart Association Class IV). (2) History of cardiovascular or cerebrovascular disease within the previous 3 months. (3) Severe arrhythmias, such as atrial flutter, atrial fibrillation, and frequent ventricular tachycardia. (4) Active malignancies with a life expectancy of less than 5 years. (5) Any condition preventing acceptable technical quality of arterial stiffness monitoring. The protocol received Institutional ethics approval and all participants provided informed consent.

Patients’ medical history, medication history, smoking history, and biochemical test indicators were collected. Biochemical test indicators included: serum total cholesterol (TC), triglyceride (TG), fasting blood glucose, high-density lipoprotein cholesterol (HDL-c), low-density lipoprotein cholesterol (LDL-c). Definition of smoking was at least one cigarette a day for more than 6 months.

All participants underwent routine physical examinations, including height, weight, heart rate (HR), waist circumference (WC), and hip circumference (HC), performed by a trained person using the same tape and platform scale. During the measurement, the subjects took off their shoes, hat and heavy coats, and stood on a platform at attention posture. BMI was calculated as BMI = weight (kg)/${\mathrm{height}}^{2}$ ($m^{2}$). WC was measured at the umbilical level circumference, and the HC was the horizontal circumference of the most prominent posterior part of the hip. Waist-hip ratio (WHR) was calculated as (waist circumference/hip circumference) × 100%. Waist-height ratio (WHtR) was calculated as (waist circumference/height) × 100%.

According to the Guidelines for Prevention and Control of Overweight and Obesity in Chinese Adults [11], the research subjects were divided into the following three groups: normal BMI group (BMI <24 kg/$m^{2}$), overweight group (24 kg/$m^{2}$≤ BMI < 28 kg/$m^{2}$), and obesity group (BMI ≥28 kg/$m^{2}$). Furthermore, according to the 2013 edition of Guidelines for Prevention and Treatment of Type 2 Diabetes in China [12], which defined central obesity (visceral obesity) as waist circumference (WC) ≥90 cm or waist-hip ratio (WHR) ≥0.90 in male, and WC ≥85 cm or WHR ≥0.85 in female, the subjects were divided into central obesity group and non-central obesity group.

After the participants sat quietly for 5 min, brachial artery blood pressure was measured by an electronic sphygmomanometer (HEM907, Omron, Kyoto, Japan) 3 times, at intervals of at least 1 min. Peripheral systolic blood pressure (PSP), peripheral diastolic blood pressure (PDP), and peripheral pulse pressure (PPP) were recorded. Peripheral mean arterial pressure (p-MAP) was calculated as (PSP + 2 × PDP)/3.

The diagnosis of hypertension is based on the criteria given in the 2010 Guidelines for Prevention and Treatment of Hypertension in China [13], which is systolic blood pressure ≥140 mmHg and/or diastolic blood pressure ≥90 mmHg and/or a previous history of hypertension with current antihypertensive therapy.

### 2.2 Indices of Central Hemodynamics and Arterial Stiffness

A pulse wave analysis (PWA) instrument (SphygmoCor-px V8.0, AtCor Medical, New South Wales, Australia) was used for measurement of central aortic pressure and PWV. Participants were in the supine position, with the right upper limb outreaching horizontally at a 45-degree angle to the body. The instrument’s contact probe was placed on the right radial artery where the pulse is strongest, and a continuous radial pulse group of at least 12 s was recorded in real time, translated into central aortic pulse wave by the computer conversion function, which determined the central aortic systolic pressure (CSP), central diastolic pressure (CDP), central pulse pressure (CPP), central mean arterial pressure (c-MAP), central augmentation pressure (CAP), central augmentation index (cAIx), and cAIx@HR75 (cAIx adjusted for 75 heartbeats/min). AIx was defined as the augmentation pressure (CAP) of the central aortic pressure waveform expressed as a percentage of CPP. Pressure pulse amplification was characterized both as the percentage ratio of PPP/CPP and by the difference between PSP and CSP.

Cf-PWV was measured by two trained study personnel using applanation tonometry with a Millar transducer and SphygmoCor CVMS system (AtCor Medical PtyLtd, Sydney, Australia). cf-PWV measurement was performed by sequential placement of the transducer on the femoral artery and carotid artery and determining transit time between the two pulses in reference to the R wave of the ECG. cf-PWV was calculated as the measured distance from the suprasternal notch to the femoral artery minus the distance from the suprasternal notch to the carotid artery divided by the pulse transit time [PWV = distance(m)/transit time(s)] by the integrated software, which automatically processed each set of pulse waves and ECG data.

According to the European Society of Hypertension expert consensus on cf-PWV for clinical practice, cf-PWV ≥10 m/s was defined as abnormal.

## 3. Statistical Analysis

SPSS 26.0 software package (SPSS, Chicago, IL, USA) and Excel were used for statistical analysis. Continuous variables are expressed as mean ± SD, categorical variables are given as frequencies and percentages. One-way ANOVA is used for the comparison of quantitative data between the 3 groups of BMI class, and chi-square test for categorical variables. The correlation between different obesity phenotypes and central hemodynamic indices was analyzed by Pearson correlation analysis. Multivariable linear regression analysis was used to identify the factors influencing cf-PWV. Stepwise multivariate linear regression was conducted to investigate the association of the different obesity assessments with cf-PWV. Furthermore, predictive value of the four obesity indicators to cf-PWV was assessed using receiver operating characteristic (ROC) curve analysis. The difference between areas under the curves (AUC) was tested using the MedCalc software (MedCalc Software Ltd, Ostend, Belgium). A two-sided *p* value < 0.05 was considered statistically significant.

## 4. Results

### 4.1 Population Characteristics

A total of 634 participants was studied, including 393 males (62.0%). The mean age of the enrolled population was (52.03 ± 12.93) years, and the mean BMI was (25.59 ± 3.93) kg/$m^{2}$. There were statistically significant differences in age, gender, WC, HC, WHtR and WHR among the BMI classes (*p *< 0.05). The three groups differed in prevalence of smoking, the overweight group had more antihypertensive therapy, and subjects were younger and more frequently male in the obesity group (*p *< 0.05), but heart rate (HR) was similar (*p* = 0.299) among BMI groups.

In the general population, there were statistically significant differences in PSP, PDP, CSP, CDP, CAP, cAIx, PPP/CPP and PSP-CSP among the three BMI groups (*p *< 0.01), but no significant differences in PPP and CPP (*p *> 0.05). Brachial and central Systolic blood pressure/Diastolic blood pressure (SBP/DBP) and cf-PWV were higher with increasing BMI (*p *< 0.05). The higher BMI groups showed lower CAP and cAIx, and PSP-CSP and PPP/CPP significantly increased with increased BMI (*p *< 0.01). TG and HDL-c of the three groups showed statistically significant differences (*p *< 0.001), while TC, LDL-c and fasting blood glucose showed no significant differences (*p *> 0.05) (Table 1).

**Table 1.** **General characteristics and haemodynamic indices of the subjects**.

	Total	Normal weight	Overweight	Obese	*p*-value
		BMI <24 kg/$m^{2}$	24 kg/$m^{2}$ ≤ BMI ≤ 28 kg/$m^{2}$	BMI ≥28 kg/$m^{2}$	
N	634	230	256	148	
Men (%)	393 (62.0%)	102 (44.3%)	180 (70.3%)	111 (75%)	<0.001
Age, y	52.03 ± 12.93	52.56 ± 12.95	52.93 ± 11.49	49.64 ± 12.35	0.025
Smoker (%)	114 (18.0%)	24 (10.4%)	57 (22.3%)	33 (22.3%)	0.001
Antihypertensive treatment (%)	254 (40%)	68 (29.6%)	118 (46.1%)	68 (45.9%)	<0.001
ACEI/ARB	144 (18.0%)	27(11.7%)	55 (21.5%)	32 (21.6%)	0.008
Bata-blockers	21 (3.3%)	4(1.7%)	13 (5.1%)	4 (2.7%)	0.133
Calcium Antagonists	88 (13.9%)	14 (6.1%)	40 (15.6%)	34 (23.0%)	<0.001
Diuretics	2 (0.3%)	1 (0.4%)	0 (0%)	1 (0.7%)	0.518
TG, mmol/L	2.00 ± 1.76	1.55 ± 0.91	2.10 ± 1.58	2.55 ± 2.66	<0.001
TC, mmol/L	4.88 ± 1.23	4.88 ± 1.05	4.92 ± 1.13	4.83 ± 1.24	0.761
HDL-C, mmol/L	1.15 ± 0.39	1.26 ± 0.42	1.09 ± 0.32	1.08 ± 0.43	<0.001
LDL-C, mmol/L	3.21 ± 0.84	3.19 ± 0.89	3.26 ± 0.89	3.18 ± 0.84	0.591
Glucose, mmol/L	5.79 ± 1.81	5.54 ± 1.63	5.89 ± 1.87	5.99 ± 1.95	0.039
Height, cm	167.29 ± 8.65	166.07 ± 8.32	167.91 ± 7.86	169.65 ± 9.69	<0.001
Weight, kg	71.97 ± 14.21	59.73 ± 7.97	73.59 ± 7.64	88.73 ± 12.21	<0.001
BMI, Kg/$m^{2}$	25.59 ± 3.93	21.85 ± 1.73	25.94 ± 1.19	30.79 ± 3.16	<0.001
WC, cm	91.18 ± 10.69	83.04 ± 8.11	92.10 ± 8.71	102.24 ± 8.71	<0.001
HC, cm	97.95 ± 7.15	92.76 ± 4.97	98.37 ± 4.79	105.26 ± 6.73	<0.001
WHtR, %	0.55 ± 0.06	0.50 ± 0.05	0.55 ± 0.04	0.60 ± 0.05	<0.001
WHR, %	0.93 ± 0.08	0.90 ± 0.08	0.94 ± 0.07	0.97 ± 0.06	<0.001
PSP, mmHg	131.67 ± 17.79	128.08 ± 18.46	133.14 ± 17.64	134.69 ± 17.79	0.001
PDP, mmHg	76.38 ± 12.29	72.97 ± 12.48	77.63 ± 11.93	79.53 ± 11.38	<0.001
PPP, mmHg	55.29 ± 12.92	55.12 ± 13.91	55.52 ± 12.09	55.16 ± 12.82	0.936
CAP, mmHg	11.61 ± 7.34	12.45 ± 7.52	11.95 ± 7.23	9.72 ± 6. 83	0.001
HR, beat/min	69.67 ± 10.53	69.62 ± 10.99	69.07 ± 9.98	70.76 ± 10.73	0.299
cAIx, mmHg	25.78 ± 12.13	27.50 ± 12.26	26.28 ± 11.94	22.24 ± 11.59	<0.001
cAIx@HR75, mmHg	23.25 ± 11.75	24.96 ± 11.25	23.42 ± 10.94	20.25 ± 10.02	<0.001
CSP, mmHg	120.03 ± 17.75	117.17 ± 18.40	121.68 ± 17.84	121.64 ± 16.03	0.009
CDP, mmHg	77.61 ± 12.50	74.21 ± 12.75	78.84 ± 12.15	80.76 ± 11.49	<0.001
CPP, mmHg	42.42 ± 11.99	42.97 ± 12.81	42.84 ± 11.51	40.87 ± 11.40	0.197
PPP/CPP (%)	1.33 ± 0.16	1.31 ± 0.16	1.32 ± 0.16	1.37 ± 0.16	<0.001
PSP-CSP, mmHg	11.63 ± 5.37	10.91 ± 5.24	11.46 ± 5.21	13.05 ± 5.61	0.001
cf-PWV, m/s	8.30 ± 1.97	8.02 ± 2.06	8.35 ± 1.88	8.62 ± 1.93	0.012

Data are mean ± SD or percentage as marked. *p*-value: independent *t*-test analysis of variance for numeric variables and chi-square test for categoric variables. TG, triglyceride; TC, total cholesterol; HDL-c, high density lipoprotein cholesterol; LDL-c, low density lipoprotein cholesterol; BMI, body mass index; WC, waist circumference; HC, hip-circumference; WHR, waist–hip ratio; WHtR, waist–height ratio; HR, Heart rate; PSP, peripheral systolic blood pressure; PDP, peripheral diastolic blood pressure; PPP, peripheral pulse pressure; CSP, central aortic systolic pressure; CDP, central diastolic pressure; CPP, central pulse pressure; CAP, central augmentation pressure; cAIx, central augmentation index; cAIx@HR75, cAIx adjusted to heart rate of 75 bpm; cf-PWV, carotid-femoral pulse wave velocity; ACEI/ARB, Angiotensin-Converting Enzyme inhibitors/Angiotensin II Receptor Antagonists.

### 4.2 Correlation between Different Obesity Assessment Phenotype and Hemodynamic Indexes

In the overall study population, using BMI, WC, WHtR and WHR as continuous independent variables, PSP, PDP, and CDP, cf-PWV were all positively associated with each one (*p *< 0.05), whereas CPP and PPP were not correlated with any obesity assessment phenotypes. CSP was positively correlated with BMI, WC and WHtR (*r* = 0.134, *p *< 0.001; *r* = 0.096, *p* = 0.016; *r* = 0.148, *p *< 0.001, respectively), but not with WHR (r = 0.069, *p* = 0.083). WHtR and WHR were positively correlated with cf-PWV (*r* = 0.267, *r* = 0.258, *p *< 0.001, respectively). BMI was positively correlated with WC, WHtR and WHR (*r* = 0.701, *r* = 0.691, *r* = 0.363, *p *< 0.001, respectively). CAP and cAIx were negatively related with BMI and WC (*p *< 0.01), while PSP-CSP was positively related with BMI (*p *< 0.01) (Table 2).

**Table 2.** **Correlation between different obesity phenotypes and central hemodynamic indexes in the total population**.

	BMI		WC		WHtR		WHR	
	*r*	*p*	*r*	*p*	*r*	*p*	*r*	*p*
PSP	0.176^**^	<0.001	0.161^**^	<0.001	0.179^**^	<0.001	0.105^**^	0.008
PDP	0.244^**^	<0.001	0.207^**^	<0.001	0.190^**^	<0.001	${\mathrm{0.087}}^{*}$	0.028
p-MAP	0.198^**^	<0.001	0.149^**^	<0.001	0.164^**^	<0.001	0.063	0.112
PPP	0.01	0.792	0.025	0.532	0.066	0.097	0.061	0.124
HR	0.075	0.06	0.061	0.125	0.073	0.065	0.01	0.796
CSP	0.134^**^	0.001	${\mathrm{0.096}}^{*}$	0.016	0.148^**^	<0.001	0.069	0.083
CDP	0.241^**^	<0.001	0.200^**^	<0.001	0.184^**^	<0.001	${\mathrm{0.080}}^{*}$	0.044
c-MAP	0.198^**^	<0.001	0.149^**^	<0.001	0.164^**^	<0.001	0.063	0.112
CPP	–0.053	0.181	–0.067	0.094	0.027	0.49	0.018	0.643
PPP/CPP	0.145^**^	<0.001	0.194^**^	<0.001	0.058	0.143	0.064	0.109
PSP-CSP	0.141^**^	<0.001	0.216^**^	<0.001	0.104^**^	0.009	0.119^**^	0.003
CAP	–0.112^**^	0.005	–0.181^**^	<0.001	–0.042	0.289	–0.06	0.13
cAIx	–0.134^**^	0.001	–0.209^**^	<0.001	–0.071	0.075	${\mathrm{–0.090}}^{*}$	0.024
cAIx@75	–0.113^**^	0.005	–0.206^**^	<0.001	–0.045	0.256	${\mathrm{–0.098}}^{*}$	0.014
cf-PWV	0.121^**^	0.002	0.217^**^	<0.001	0.267^**^	<0.001	0.258^**^	<0.001
BMI	1		0.701^**^	<0.001	0.691^**^	<0.001	0.363^**^	<0.001

PSP, peripheral systolic blood pressure; PDP, peripheral diastolic blood pressure; PPP, peripheral pulse pressure; p-MAP, peripheral mean arterial pressure; CSP, central aortic systolic pressure; CDP, central diastolic pressure; c-MAP, central mean arterial pressure; CPP, central pulse pressure; CAP, central augmentation pressure; cAIx, central augmentation index; cAIx@HR75, cAIx adjusted to heart rate of 75 bpm; cf-PWV, carotid-femoral pulse wave velocity. BMI, body mass index; WC, waist circumference; HC, hip-circumference; WHR, waist–hip ratio; WHtR, waist–height ratio; HR, Heart rate. *p < 0.05; **p < 0.01.

### 4.3 Consistency between Obesity Diagnosed by BMI and Central Obesity Assessed by WC and WHR.

Participants were divided into three groups according to the BMI compared with groups with central obesity assessed by WC and WHR. We found that the results of the two groups were relatively consistent with the obese BMI class agreeing with higher WC and WHR in males reaching 98.2%, but less (94.6%, 91.9% respectively) in females (Tables 3,4).

**Table 3.** **Consistency of central obesity in different BMI subgroups in females**.

	Normal weight	Overweight	Obese	Total N = 241	$c^{2}$	*p*-value
	BMI <24 kg/$m^{2}$	24 kg/$m^{2}$ ≤ BMI < 28 kg/$m^{2}$	BMI ≥28 kg/$m^{2}$			
WC <85 cm	93 (72.7%)	11 (14.5%)	2 (5.4%)	106 (44.0%)	91.924	<0.001
WC ≥85 cm	35 (27.3%)	65 (85.5%)	35 (94.6%)	135 (56.0%)		
WHR <0.85	52 (40.6%)	8 (10.5%)	3 (8.1%)	63 (26.1%)	29.737	<0.001
WHR ≥0.85	76 (59.4%)	68 (89.5%)	34 (91.9%)	178 (73.9%)		

BMI, body mass index; WC, waist circumference; WHR, waist–hip ratio.

**Table 4.** **Consistency of central obesity in different BMI subgroups in males**.

	Normal weight	Overweight	Obese	Total N = 393	$c^{2}$	*p*-value
	BMI <24 kg/$m^{2}$	24kg/$m^{2}$ ≤ BMI < 28 kg/$m^{2}$	BMI ≥28 kg/$m^{2}$			
WC <90 cm	71 (69.6%)	49 (27.2%)	2 (1.8%)	122 (31.0%)	116.43	<0.001
WC ≥90 cm	31 (30.4%)	131 (72.8%)	109 (98.2%)	271 (69.0%)		
WHR <0.9	71 (69.6%)	49 (27.2%)	2 (1.8%)	122 (31.0%)	116.43	<0.001
WHR ≥0.9	31 (30.4%)	131 (72.8%)	109 (98.2%)	271 (69.0%)		

BMI, body mass index; WC, waist circumference; WHR, waist–hip ratio.

### 4.4 Linear Regression Analysis of the Relationship between Different Obesity Assessments and cf-PWV

Multiple linear regression analysis was performed to evaluate the independent risk factors of cf-PWV. After adjusting for age, sex, heart rate, antihypertensive therapy, blood pressure, glucose and LDL-c, when BMI, WC, WHtR, WHR were separately put into the model, BMI was not an independent risk factor for cf-PWV (β= 0.044, *p* = 0.22), but WC, WHtR and WHR were independent risk factors for cf-PWV (β = 0.120, *p* = 0.001, β= 0.103, *p* = 0.004, β= 0.092, *p* = 0.013) (Table 5). However, in the stepwise linear regression model, together with cardiovascular risk factors, only WC was significantly associated with cf-PWV (β= 0.135, *p *< 0.001).

**Table 5.** ** Multivariate linear regression analysis of independent risk factors of cf-PWV**.

		B	Std. error	Beta	*p*-value	$R^{2}$
Model 1						0.403
	Sex	0.267	0.141	0.067	0.058	
	Age	0.069	0.006	0.429	<0.001	
	PSP	0.036	0.004	0.328	<0.001	
	HR	0.016	0.006	0.085	0.014	
	Antihypertensive treatment	0.181	0.137	0.046	0.188	
	LDL-c	–0.034	0.078	–0.015	0.659	
	Glucose	0.144	0.038	0.13	<0.001	
	BMI	0.022	0.018	0.044	0.22	
Model 2						0.413
	Sex	0.119	0.148	0.030	0.420	
	Age	0.068	0.006	0.424	<0.001	
	PSP	0.035	0.004	0.322	<0.001	
	HR	0.015	0.006	0.081	0.018	
	Antihypertensive treatment	0.175	0.136	0.045	0.198	
	LDL-c	–0.045	0.077	–0.020	0.561	
	Glucose	0.127	0.038	0.115	0.001	
	WC	0.022	0.007	0.120	0.001	
Model 3						0.411
	Sex	0.263	0.138	0.066	0.057	
	Age	0.066	0.006	0.413	<0.001	
	PSP	0.035	0.004	0.322	<0.001	
	HR	0.015	0.006	0.080	0.021	
	Antihypertensive treatment	0.169	0.136	0.043	0.215	
	LDL-c	–0.042	0.077	–0.018	0.583	
	Glucose	0.128	0.038	0.116	0.001	
	WHtR	3.521	1.203	0.103	0.004	
Model 4						0.408
	Sex	0.187	0.145	0.047	0.197	
	Age	0.066	0.006	0.411	<0.001	
	PSP	0.036	0.004	0.332	<0.001	
	HR	0.016	0.006	0.086	0.013	
	Antihypertensive treatment	0.179	0.136	0.045	0.190	
	LDL-c	–0.040	0.077	–0.018	0.603	
	Glucose	0.127	0.039	0.115	0.001	
	WHR	2.312	0.924	0.092	0.013	

cf-PWV, carotid-femoral pulse wave velocity; LDL-c, low density lipoprotein cholesterol; BMI, body mass index; HR, Heart rate; WC, waist circumference; WHR, waist–hip ratio; WHtR, waist–height ratio; PSP, peripheral systolic blood pressure.

### 4.5 Diagnostic Value of Different Obesity Assessment Indicators for Vascular Stiffness

**Fig. 1.**
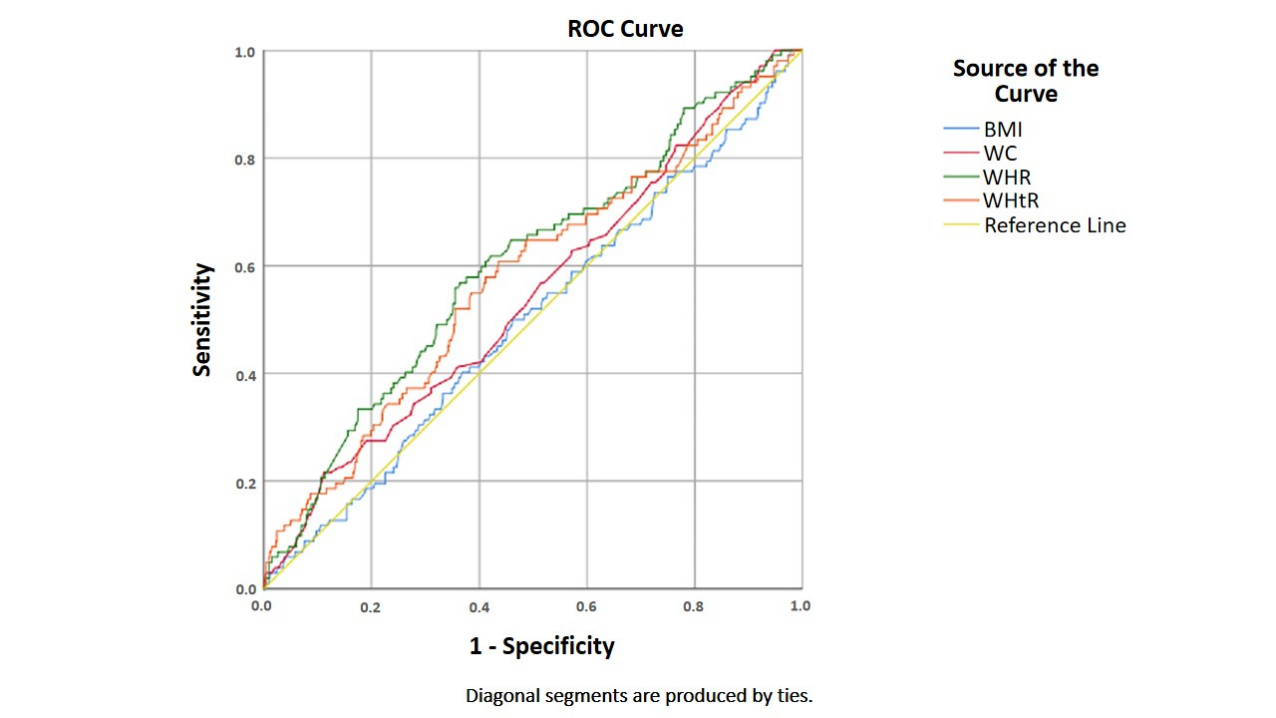
**ROC curves of PWV predicted by different obesity indicators**. WC (AUC = 0.545, *p* = 0.151); BMI (AUC = 0.500, *p* = 0.992); WHtR (AUC = 0.577, *p* = 0.013); WHR (AUC = 0.603, *p* = 0.001); AUC, Area Under the receiver operating characteristic curve.

Regarding cf-PWV ≥10 m/s as the gold standard of vascular stiffness, the receiver operating characteristic (ROC) curve of BMI, WC, WHR and WHtR is shown in Fig. 1 WC (AUC = 0.545, *p* = 0.151); BMI (AUC = 0.500, *p* = 0.992); WHtR (AUC = 0.577, *p* = 0.013); WHR (AUC = 0.603, *p* = 0.001). The difference between areas under the curves (AUC) was tested using the MedCalc software. There was a difference in the area under the curve between WHR and BMI, (Z = 2.312, *p* = 0.021), no significant difference between WHR, WHtR and WC (*p *> 0.05), and the area under ROC curve (AUC) of WHR was greater than BMI (*p *< 0.05).

## 5. Discussion

In this study, we found that brachial and central SBP and DBP and cf-PWV were higher with increasing BMI, as were PSP-CSP and PPP/CPP. The higher BMI groups showed lower CAP and cAIx. BMI had inconsistencies with fat distribution indicators in obesity diagnosis, especially in females, and was different from the other obesity-related metabolic phenotypes. After all risk factors were adjusted, only WC was found to be an independent risk factor for cf-PWV. The ROC curve showed that WHR may have greater predictive value for vascular stiffness than BMI, but there was no significant difference between WHR, WHtR and WC. Although all of the correlations between arterial stiffness measures and obesity variables are significant, they are weak, with r values < 0.2 in Table 2. Fig. 1. is consistent with this finding, showing the largest C-index to be 0.60, which is not robust.

Although some studies have indicated that a higher BMI is frequently accompanied by hypertension, dyslipidemia and endothelial dysfunction [14, 15, 16], some individuals with increased BMI show a decreased risk of mortality [17, 18, 19], a phenomenon that has been called the “obesity paradox”. Our study also showed that in the general or male population, BMI was negatively correlated with CAP and cAIx, and positively correlated with PPP/CPP and CSP. That is, as BMI increases, the amplifying effect of pulse pressure makes male blood vessels appear younger, suggesting that obese people may have better arterial compliance than normal-weight people. In addition, BMI was not found to be an independent predictor for PWV after all risk factors were adjusted. These results were consistent with the obesity paradox to some extent.

These paradoxical results may be due, partly at least, to a limitation of BMI. It is well known that BMI was developed as a measure of weight rather than an index of obesity [20, 21], which may make it misleading in the estimation of body fat content. In our study, the difference between BMI and fat distribution indicators in diagnosing obesity was significant, which suggested BMI should not be used as a core index to evaluate central obesity.

Although BMI has been widely used to measure adiposity in many countries, including Asians, the American Heart Association (AHA) recommended in 2015 that the waist circumference should be used to assess the risk of cardiovascular diseases in Asians, partly because of the low sensitivity of BMI for cardiovascular risk [22]. In patients with coronary heart disease, there was no obesity paradox when body fat ratio (BF%) was used to replace BMI. BF% was associated with a higher risk of major adverse cardiovascular events (MACE), while fat-free mass was associated with a lower risk of MACE, suggesting that BMI was not associated with MACE [23].

A growing body of evidence suggests that fat distribution may be more important than overall adiposity. For instance, visceral fat is a strong and independent predictor of metabolic disorders, such as dyslipidemia, insulin resistance and type 2 diabetes [24, 25, 26]. Conversely, subcutaneous fat may have a beneficial effect on metabolism [27]. Increased visceral to subcutaneous fat area ratio (VSR) was an independent predictor of all-cause mortality, suggesting that the location of fat deposits may be more important than actual body fat mass [28]. In this study, using cf-PWV as a gold standard for vascular stiffness, we found that WC, WHtR and WHR were independent predictors of cf-PWV. In multivariate stepwise linear regression, WC was the strongest predictor for vascular stiffness. BMI, however, was not a predictor. Furthermore, when cf-PWV ≥10 m/s was used as the standard for vascular stiffness, the results showed that WHR had better predictive value than did BMI.

Some previous studies [29, 30] have found a positive correlation between BMI and PWV, but that blood pressure was the most powerful predictor for PWV. Therefore, after adjusting for cardiovascular risk factors, especially blood pressure, some clinical studies have found no significant correlation or even a negative correlation between BMI and PWV [31, 32, 33]. The reason may be related to the different detection methods of PWV and the difference in the selected pulse wave travel distance. In addition, obese patients with excessive diabetes, hypertension, cardiovascular risk factors and other risk factors may appropriately weaken the correlation between PWV and BMI.

This study has some limitations: (i) As a cross-sectional study with a small sample size, the results need to be further confirmed in prospective studies. (ii) In this study, obesity types were grouped according to BMI, waist circumference, hip circumference and waist-to-hip ratio, without considering different fat distribution and body fat rate, or obesity types associated with metabolic abnormalities. Umbilical cord plane CT scan is currently recognized as the gold standard for visceral fat measurement, but visceral fat was not measured in this study. (iii) Our results show that all obesity measures are weakly associated with atherosclerosis. (iv) The large proportion of people receiving antihypertensive drugs and different antihypertensive drugs may cause possible confounding effects. (v) Blood pressure was taken as the average of three measurements; it is likely inflated by the first value due to initial stimulus or short resting period. It may be better to average the second and third measures. And the cuff sphygmomanometer is cylindrical rather than conical, which may be more appropriate for obese participants with large upper arms. (vi) The study was conducted in an Asian population, and it is not known whether the results will hold true for other ethnic groups.

## 6. Conclusions

The higher BMI groups showed lower CAP and cAIx. PSP-CSP and PPP/CPP were also highest in the obese group. BMI had poor consistency with fat distribution indicators in obesity diagnosis, especially in females. After adjusting for all cardiovascular risk factors, only WC was found to be an independent risk factor for cf-PWV. WHR may have greater predictive value for vascular stiffness than other indices of obesity.

## Abbreviations

BMI, body mass index; WC, waist circumference; HC, hip circumference; WHR, waist–hip ratio; WHtR, waist–height ratio; BP, blood pressure; HR, Heart rate; PSP, peripheral systolic blood pressure; PDP, peripheral diastolic blood pressure; PPP, peripheral pulse pressure; p-MAP, peripheral mean arterial pressure; CSP, central aortic systolic pressure; CDP, central diastolic pressure; c-MAP, central mean arterial pressure; CPP, central pulse pressure; CAP, central augmentation pressure; cAIx, central augmentation index; cAIx@HR75, cAIx adjusted to heart rate of 75 bpm; cf-PWV, carotid-femoral pulse wave velocity. TG, triglyceride; TC, total cholesterol; HDL-C, high density lipoprotein cholesterol; LDL-C, low density lipoprotein cholesterol.
